# The Expert Patient Clinic (QI Project): A Meaningful New Community Psychiatry Training Experience for Medical Students

**DOI:** 10.1192/bjo.2024.301

**Published:** 2024-08-01

**Authors:** Jason Holdcroft-Long, Natalie Dean, Meg Rayner, Elizabeth Mullins

**Affiliations:** 1 Derbyshire Healthcare NHS Foundation Trust, Derby, United Kingdom; 2 University of Nottingham, Nottingham, United Kingdom

## Abstract

**Aims:**

Psychiatry is predominantly a community specialty, but large medical school cohorts and limited outpatient learning opportunities mean students report a lack of experience in community mental healthcare. They describe clinicians lacking time to teach in busy clinics, or patients declining student presence. Consequently, many Foundation Doctors will first experience working with outpatients when they sit down to their first clinic! Our aim, quite simply, was to remedy this gap.

**Methods:**

The Psychiatry Teaching Unit at Derbyshire Healthcare is in the vanguard of patient involvement, with a large group of Expert Patients (EPs) having extensive lived experience of inpatient/outpatient psychiatric care, and medical education delivery.

We co-produced an Expert Patient Clinic to replicate a psychiatric outpatient clinic, with students acting as psychiatrists, reviewing Expert Patients. Students work in groups, taking turns as doctor/observer. Each ‘appointment’ is followed by tailored feedback.

The tasks are themed as follows:

Patient-specific review: a more ‘technical’ task e.g. reviewing medication changes and side effects, or using measurement tools to assess signs and symptoms.

Psychosocial review: considering social circumstances, activities of daily living and personal functioning.

Current mental health review: assessing mental state, subjective and objective signs and symptoms of mental health problems, and concerns, ideas and expectations for care and intervention.

Sessions are facilitated by a psychiatrist, Lived Experience Facilitator (EPs formally employed as educators) and a senior clinical nurse educator.

Pre- and post-session we ask students to assess/rate their confidence and competence in reviewing outpatients, discussing risk, and planning care, in an outpatient appointment.

**Results:**

Results so far are overwhelmingly positive with both written and numerical feedback acknowledging a significant improvement in student confidence and self-rated competence across the board. A chart in our poster shows the large increases in self-rated Likert scales measuring aspects outlined above. Qualitative verbal feedback outlines the value of having a session with real patients where they can try consultation techniques and receive instant feedback, and learning through discussing with EPs their individual stories and clinical histories. Accounts from EPs document their own learning from the sessions and development of skills in giving feedback.

**Conclusion:**

The EP Clinic provides an opportunity for students to experience clinical responsibility and practise in a safe environment with real patients. It provides valuable, realistic and high quality experience in community psychiatry without the disappointments often unavoidable in live clinical services.

